# Acute lymphoblastic leukemia relapse presenting as retinal vasculitis

**DOI:** 10.1002/ccr3.2895

**Published:** 2020-05-08

**Authors:** Imen Ksiaa, Melek Kechida, Sourour Zina, Marouane Lahdhiri, Nesrine Abroug, Moncef Khairallah

**Affiliations:** ^1^ Department of Ophthalmology Faculty of Medicine Fattouma Bourguiba University Hospital University of Monastir Monastir Tunisia; ^2^ Department of Internal Medicine and Endocrinolgy Faculty of Medicine Fattouma Bourguiba University Hospital University of Monastir Monastir Tunisia

**Keywords:** acute medicine, emergency medicine, hematology, ophthalmology

## Abstract

Retinal vasculitis may occur as an isolated manifestation of acute lymphoblastic leukemia (ALL) relapse and precede central nervous involvement. Therefore, a high index of suspicion and repeated ocular and neurological evaluations are essential for early diagnosis and prompt appropriate treatment to save life and sight.

## Graphical abstract

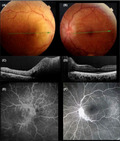

## 1 BACKGROUND

To report a case of acute lymphoblastic leukemia (ALL) relapse presenting as retinal vasculitis. A 33‐year‐old man with a history of T‐cell ALL with complete remission presented with bilateral blurred vision. A diagnosis of bilateral idiopathic vasculitis was made based on a negative work‐up for T‐cell ALL relapse and for other infectious or noninfectious etiologies. The patient was treated with oral steroids. Six months later, he presented with severe vision loss in the right eye (no light perception). Fundus examination showed features of optic nerve infiltration associated with a combined central retinal artery and vein occlusion in the right eye and optic disk swelling in the left eye. Magnetic resonance imaging of the brain and orbits and cerebrospinal fluid examination showed no infiltrative features. Peripheral blood and bone marrow studies showed no blastic cells. Results of a second MRI brain evaluation were normal, but cerebrospinal fluid analysis revealed blastic cells. The patient was referred to his hematologist for new courses of intravenous and intrathecal chemotherapy. Isolated ocular involvement in the form of retinal vasculitis progressing to infiltrative optic neuropathy may reveal disease relapse in patients with T‐cell ALL. Early diagnosis is of utmost importance for prompt initiation of appropriate therapy to save life and sight.

Optic nerve and retinal manifestations associated with acute lymphoblastic leukemia (ALL) are frequent and mostly asymptomatic. Severe visual loss, however, can occur mainly due to severe optic nerve infiltration, ischemic optic neuropathy, and increased intracranial pressure.^1^, ^2^ Ocular manifestations can occur during either acute phase of the disease or relapse phase.^3^

We herein describe a patient who presented with retinal vasculitis as the initial manifestation of acute lymphoblastic leukemia relapse.

## 2 CASE PRESENTATION

**FIGURE 1:**
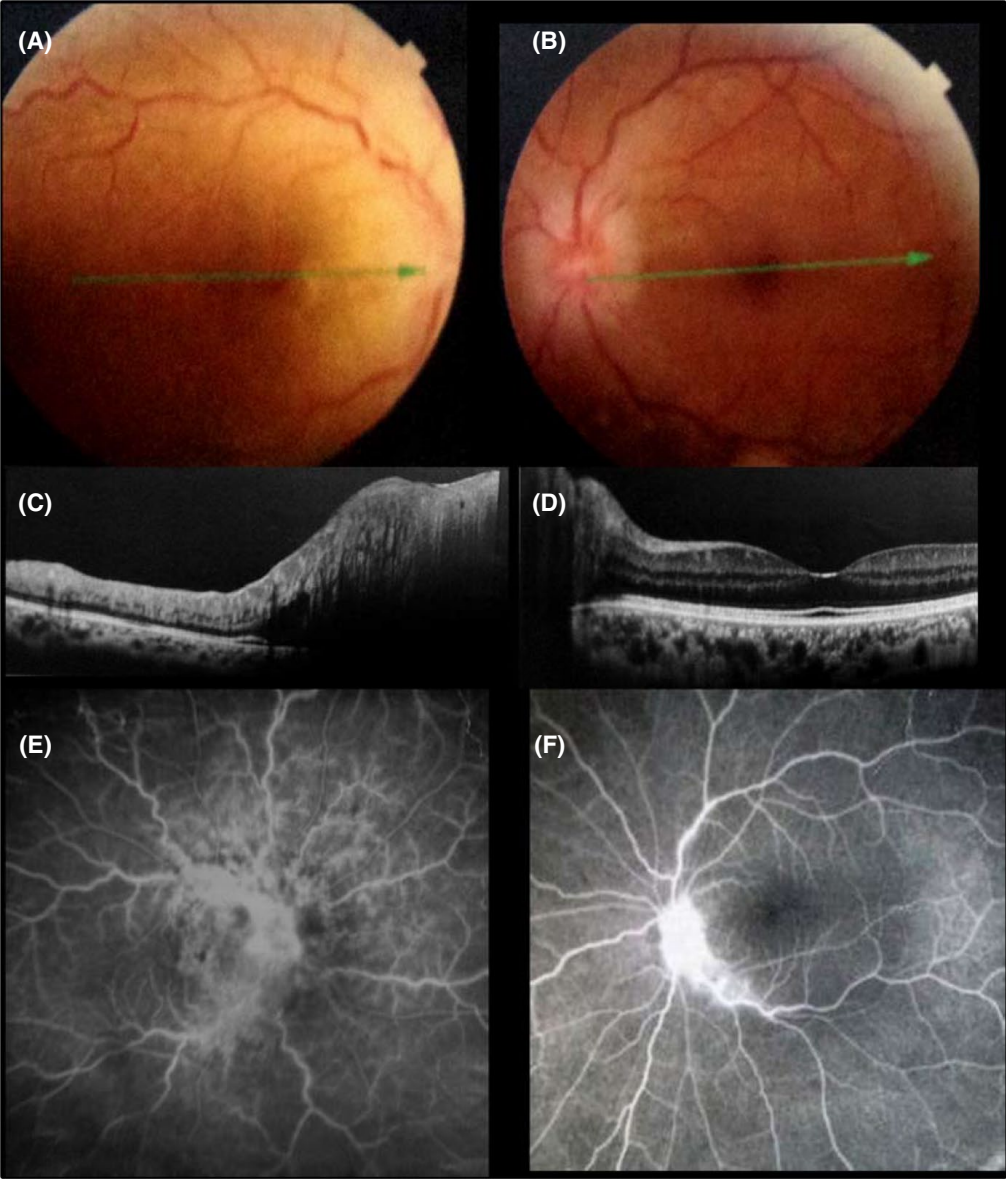
Fundus photograph (A, B) and spectral domain optical coherence tomography (C, D) showing bilateral optic disk and peripapillary retinal edema, more marked in the right eye with retinal vein dilation and tortuosity in both eyes. Fluorescein angiography (E, F) showing bilateral diffuse retinal vascular leakage and optic disk hyperfluorescence, more marked in the right eye (E, F)

A 33‐year‐old patient was diagnosed with T‐cell acute lymphoblastic leukemia on June 2015. He underwent courses of chemotherapy and allogeneic bone marrow transplantation on October 2015. Details of chemotherapy were not available. Subsequent peripheral blood smears and examination of the bone marrow documented the achievement of complete remission. No intrathecal chemotherapy for central nervous system prophylaxis was performed. When first ocular symptoms occurred, the patient was not under any immunosuppressive treatment. He complained of blurred vision in both eyes on July 2016. His visual acuity was 20/70 in the right eye (RE) and 20/20 in the left eye (LE). Fundus examination (Figure 1) and spectral domain optical coherence tomography (SD‐OCT; Figure 1) showed bilateral optic disk and peripapillary retinal edema, more marked in the RE. There also were retinal vein dilation and tortuosity in both eyes. Fluorescein angiography was performed showing bilateral diffuse retinal vascular leakage and optic disk hyperfluorescence, more marked in the RE (Figure 1). Peripheral blood cells studies, cranial and orbital MRI, and cerebrospinal fluid analysis showed no abnormal findings. Results of work‐up for underlying infectious or noninfectious diseases including clinical evaluation by internist, C reactive protein, Quantiferon test, purified protein derivative skin test, chest X‐ray, angiotensin‐converting enzyme level, urinary and blood calcium levels, syphilis serology, and HIV test were normal or negative.

A diagnosis of bilateral idiopathic retinal vasculitis was made. The patient was treated with oral corticosteroids: 80 mg of prednisone per day (patient's weight: 82 kg), followed by gradual tapering for 6 months.

On 14 December 2016, the patient experienced mild headaches. Results of neurological examination were unremarkable. A second MRI of the brain and orbits was performed and showed no abnormalities. Lumbar puncture revealed normal intracranial pressure and sample study showed no malignant cells. The patient was managed with oral paracetamol and continued steroid therapy.

**FIGURE 2:**
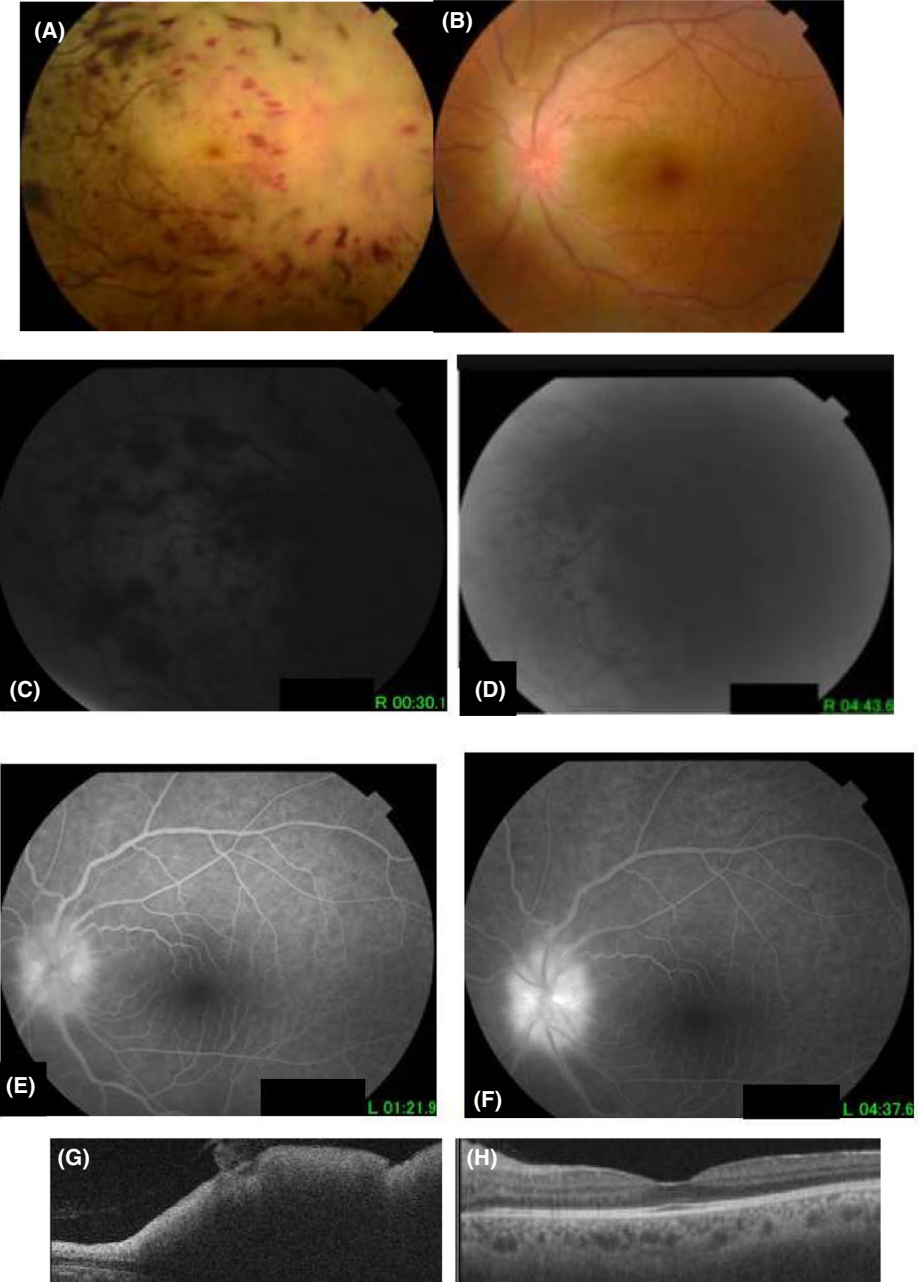
(A) Fundus photograph of the right eye taken 6 mo after initial presentation showing a marked optic disk edema with papillary and peripapillary infiltration, multiple flame‐shaped and blot and dot retinal hemorrhages, diffuse retinal whitening, and retinal venous dilation and tortuosity. (B) Fundus photograph of the left eye showing a mild retinal venous dilation and optic disk swelling. Early (C) and late (D) fluorescein angiograms of the right eye showing a masking effect from retinal hemorrhages and the lack of filling of retinal vessels. Early (E) and late (F) fluorescein angiograms of the left eye showing optic disk leakage. B‐scan OCT showing a markedly elevated optic nerve head with prominent peripapillary retinal thickening in the right eye (G) and a normal retinal thickness in the left eye (H)

He presented to our department for the first time on 27 January 2017 with a 10‐day history of reduced vision in the RE. On ophthalmological examination, there was no light perception in the RE and visual acuity was 20/20 in the LE. There was a right relative afferent pupillary defect. There were no cells in the anterior chamber or vitreous. Results of slit lamp examination were unremarkable in both eyes. Fundus examination of the RE showed a marked optic disk edema with papillary and peripapillary infiltration, multiple flame‐shaped and blot and dot retinal hemorrhages, retinal whitening, and retinal venous dilatation and tortuosity (Figure 2). Fundus examination of the LE showed a moderate optic disk edema (Figure 2). Fluorescein angiography of the RE showed absence of filling of retinal vasculature and optic disk hyperfluorescence in the LE. SD‐OCT of the RE showed a markedly elevated optic nerve head with prominent peripapillary retinal thickening (Figure 2). A diagnosis of leukemic optic nerve infiltration associated with combined central retinal artery and vein occlusion was highly suspected.

Bone marrow aspiration revealed no blastic cells. Cerebrospinal fluid analysis and orbital and cranial MRI were performed and failed to show infiltrative features.

**FIGURE 3:**
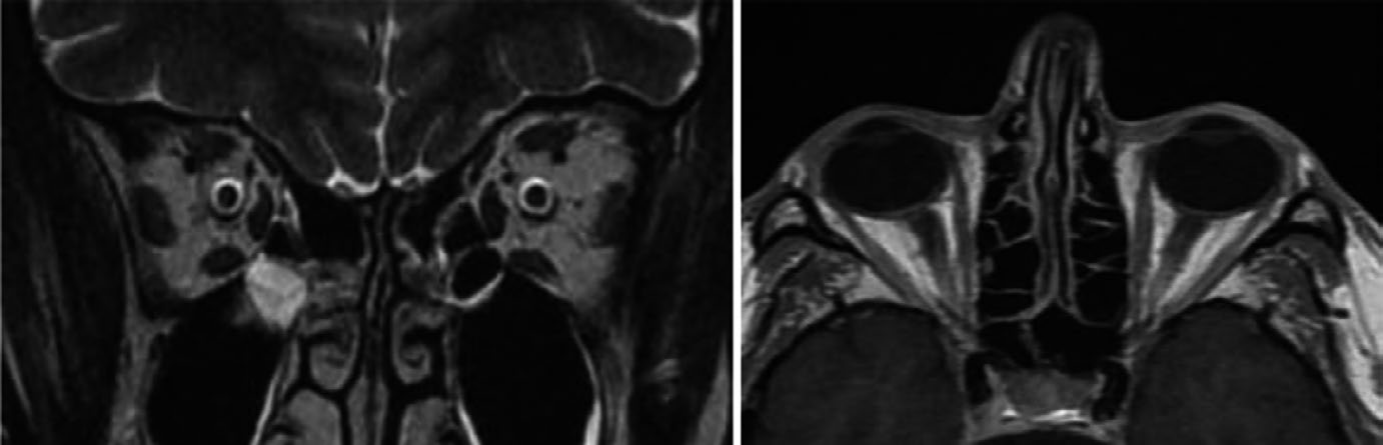
Coronal (A) and axial (B) T2 MRI images of the optic nerves showing no evidence of brain infiltration

On February 2017 further orbital and cranial MRI was performed and again failed to reveal any abnormal changes in the brain (Figure 3). A cerebrospinal fluid analysis, however, showed blastic cells at the rate of 2% concurrent with central nervous system (CNS) involvement.

The patient was referred to his hematologist for new courses of intravenous and intrathecal chemotherapy. Details of chemotherapy at this stage also were not available. However, the patient passed away 18 months after first ophthalmic complaints.

## 3 DISCUSSION

In this patient with a history of achieved remission from T‐cell ALL and no evidence of immunodepression, initial blurring of vision was considered to be caused by idiopathic retinal vasculitis. Results of peripheral blood cells studies, cranial and orbital MRI, and cerebrospinal fluid analysis showed no evident abnormal findings consistent ALL relapse. Work‐up for underlying noninfectious and infectious diseases, including tuberculosis, syphilis, and sarcoidosis, also was negative. On follow‐up, severe visual loss occurred due to marked optic nerve infiltration associated with combined central retinal artery and vein occlusion. Repeated neurological investigations allowed a diagnosis of CNS involvement to be established only 7 months after the first patient's ocular complaints.

Retinal manifestations in preexisting ALL are frequent.^2^ “Leukemic retinopathy” is a term used to describe vascular features secondary to severe anemia, thrombocytopenia, and hyperviscosity rather than to direct leukemic infiltration.^4^ Common retinal vascular manifestations associated with ALL are white‐centered retinal hemorrhages and cotton wool spots. Venous dilation and tortuosity are less common. Frosted branch angiitis, characterized by widespread translucent retinal perivascular sheathing of retinal vessels, was rarely reported in association with relapsing ALL and Hodgkin lymphoma.^5^, ^6^, ^7^

Human T‐cell lymphotropic virus type 1 (HTLV‐1) infection, which is known for its association with adult T‐cell leukemia/lymphoma, has been associated with uveitis and retinal vasculitis. The disease is mainly endemic to Japan, the Caribbean islands, and parts of Central Africa and South America.^8^, ^9^ Its prevalence elsewhere is low and limited to groups that have migrated from areas of endemicity. HTLV‐1 serology was not performed in our patient from a nonendemic region.

Optic disk swelling in ALL can result from infiltration by blastic cells or be secondary to a central retinal vein occlusion. Papilledema due to increased intracranial pressure is less common.

Retinal manifestations such as serous retinal detachment revealing a relapse of ALL are uncommon.^3^, ^4^ Although only few cases described optic nerve involvement in a relapse of acute lymphoblastic leukemia, none have described concomitant normal MRI of the orbits and cerebrospinal fluid analysis.^1^, ^10^ Our case shows that isolated ocular involvement in the form of retinal vasculitis progressing to infiltrative optic neuropathy may precede by weeks or months the confirmation of ALL relapse with CNS infiltration.

The involvement of the CNS and the ocular tissues in our patient might be caused by the poor chemotherapy penetration and the lack of intrathecal prophylactic treatment. Thus, a separate treatment modality, often radiotherapy, is required for the optic nerve involvement in leukemia.

In conclusion, retinal vasculitis may occur as an isolated manifestation of ALL relapse. A high index of suspicion, careful examination, close follow‐up with repeated ocular, hematological, and neurological evaluation are essential for early diagnosis and prompt therapy for this potentially blinding and fatal condition.

## CONFLICT OF INTEREST

None declared.

## AUTHOR CONTRIBUTION

IK and SZ: have made substantial contribution to conception and design of the manuscript as they interpreted data, drafted the work, approved the final version, and agreed to be accountable for all aspects of the work. MK and ML: have been involved in drafting the manuscript, approving the final version, and agreed to be accountable for all aspects of the work. NA: has been involved in critically revising the manuscript, approving the final version, and agreed to be accountable for all aspects of the work. MKh: has critically revised the work, given the final approval of the version to be published, and agreed to be accountable for all aspects of the work.
